# “Traditionally you would be passing them from pillar to post”: an evaluation exploring the Life Rooms model of partnership working

**DOI:** 10.1186/s12913-021-06672-1

**Published:** 2021-07-01

**Authors:** Joanne Worsley, Clare Rotheram, Rhiannon Corcoran

**Affiliations:** 1 Department of Primary Care and Mental Health, University of Liverpool, Liverpool, UK; 2 The Life Rooms, Mersey Care NHS Foundation Trust, Liverpool, UK

**Keywords:** Partnerships, Shared values, Mutuality, Reciprocity, Power, Resources

## Abstract

**Background:**

NHS systems are under increasing, unsustainable pressure. In the context of rising demand, limited resources and changing population needs, partnership working across sectors is crucial. The Liverpool City Region has a richness of voluntary organisations and community based assets that the Life Rooms innovation draws upon to widen the base of health and wellbeing support. The Life Rooms therefore aims to facilitate the collaboration of NHS systems with arts, voluntary and community organisations in the local health economy. This evaluation explores the Life Rooms model of partnership working spread over in excess of one hundred partnerships with voluntary, community, and corporate sector organisations.

**Methods:**

The evaluation drew on thematic analysis of semi-structured interviews with Life Rooms staff members (*n* = 10), partner organisations (*n* = 16), and users of the Life Rooms (*n* = 7).

**Results:**

Five overarching themes were identified: Quality of partnerships; Benefits of partnership working; Facilitators; Challenges within the Life Rooms model; and Making things even better.

**Conclusions:**

One of the significant successes of the Life Rooms partnership working model is the way in which the service collaborates with a wide range of organisations with the aim of providing more effective and holistic support. The success of this approach illustrates how, led by NHS Trust innovation, multiple bodies can play a role in supporting health care by bringing unique skills, expertise and programmes together to ensure multiple options to support the multiple different aspects of people’s health. These insights may be useful to other NHS organisations that may be considering a similar integration agenda.

## Introduction

Common mental health problems are said to affect one in six people in the United Kingdom (UK, [1]). Despite the ubiquitous nature of mental health problems, people from poorer socio-economic backgrounds are disproportionally affected [2]. Poor mental health has been associated with socio-economic factors such as poverty, unemployment, low educational attainment, and poor housing [2], and the estimated cost to the National Health Service (NHS) of socioeconomic inequality is £4.8 billion a year in greater hospitalisations alone [3].

Although the medical model of mental health dominates current practice, there has been renewed interest in social approaches to health, which take into account wider issues that affect people [4, 5]. Policy developments within the UK acknowledge the value of social approaches to health (e.g., the Five Year Forward View for Mental Health 2016; NHS Long Term Plan, 2019). According to the Five Year Forward View [6], NHS systems are under increasing pressure as people are living longer, often with complex health issues. The sustainability of the NHS is therefore reliant on a preventative approach. The Five Year Forward View highlights a number of ways in which this could be achieved including supporting people to manage their own health and wellbeing, and building strong partnerships with the voluntary and community sector [6]. Consistent with this, the NHS Long Term Plan [7] and the Community Mental Health Framework [8] also highlight the need for closer working across sectors to address the wider determinants of health. In the context of rising demand, limited resources and changing population needs, partnership working across sectors is crucial. There is an emerging body of research and theory on partnership working and collaboration. Some of the key principles of successful partnership working are: a common understanding of mutual benefit; establishing mutual trust; common or complimentary goals; sharing of information and resources with a relatively equal balance of power; joint-working where each partner is bringing different, complimentary expertise; and consistent and effective communication [9, 10]. As different providers bring different elements of expertise, working in partnership can deliver high quality care through considering more holistic options [11].

Greater collaboration between the healthcare sector and community, voluntary and third sector organisations has been promoted to help mitigate the effects of social determinants of health, especially as integration between health and the third sector has the potential to deliver improved health outcomes for communities [12, 13]. One such model is social prescribing. Social prescribing is a community-based model for health and wellbeing that has received increased attention over recent years. Recognising that a range of socio-economic factors determine people’s health, social prescribing seeks to address an individual’s needs in a holistic way by connecting them with practical and social support in their community. Although there is no widely agreed definition of social prescribing, the Social Prescribing Network has defined it as “a means of enabling GPs and other frontline healthcare professionals to refer patients to a link worker – to provide them with a face to face conversation during which they can learn about the possibilities and design their own personalised solutions, i.e., co-produce their ‘social prescription’ – so that people with social, emotional or practical needs are empowered to find solutions which will improve their health and wellbeing, often using services provided by the voluntary and community sector” ([14] p.19). Interventions often include exercise, housing, welfare and debt advice, adult education, self-help groups, and arts and cultural activities [15].

There are many different models of social prescribing; however, most involve a link worker who meets with individuals to discuss their needs and direct them to appropriate sources of support provided by local voluntary and community organisations. One example is the Rotherham Social Prescribing Service where people with long-term health conditions and/or mental health difficulties are referred to advisors who work alongside them to address their wider social needs by helping them access voluntary and community activities and services. The economic effectiveness of the Rotherham Social Prescribing Pilot has been demonstrated as patients’ use of hospital resources decreased by up to a fifth following their referral to social prescribing [16]. Patients also experienced a number of benefits such as improved mental and physical health, feeling less lonely and more engaged with their local community [16]. The effectiveness of social prescribing as a way of connecting individuals to arts-based activities for health and social outcomes has also been demonstrated. For example, an evaluation of a 12-week arts programme involving adults with mild to moderate mental health difficulties demonstrated a decrease in social isolation and a reduction in symptoms of depression and anxiety [17]. Thus, participatory creative activities can help to improve aspects of social cohesion, and reduce anxiety and depression.

### The Life Rooms, Mersey Care NHS Foundation trust

In 2016, Mersey Care NHS Foundation Trust, a specialist provider of mental health and learning disability services in North West England, introduced the Life Rooms. The Life Rooms prioritises a non-clinical and community-focused approach, working with non-NHS agencies to address social determinants of health, encourage social inclusion, and challenge the stigma attached to mental distress. The Life Rooms is coproduced as it is designed and developed in a side by side way alongside the communities it serves. This means that the service is shaped by people who access, work and volunteer within the service, as well as partners and the wider community. The Life Rooms has three main sites, known as community hubs, and two peripatetic sites within two locations (Liverpool and Sefton) in the North West of England. The North West is one of the most disadvantaged areas in the country [18], with higher rates of premature mortality, hospital admissions, and common mental health difficulties such as depression and anxiety [19]. Although the hubs are physical spaces facilitated by the NHS, these spaces belong to the community. Given the explicit recognition by an NHS organisation of the role of community in health, the Life Rooms is an innovative, unique service in the UK.

The broader aims of the Life Rooms relate to systemic change shifting focus towards the role society plays in the wellbeing of the population. Working within a social model offers opportunities to address social determinants and prevents deterioration in health. As policy developments in the UK acknowledge the value of social approaches to health and the role of community in health (e.g., [7, 8]), the Life Rooms model aligns with the national guidance. This non-clinical approach provides integration of public, private, voluntary and community sector services, and over 100 partnerships with statutory, private and voluntary sector organisations have developed as part of the Life Rooms model.

The Life Rooms is designed to support the prevention and population health agendas through a three-pillar model: social prescribing, learning and community. At the Life Rooms, social prescribing is offered through the pathways advice service. Link workers, known as pathways advisors, offer a one-to-one daily drop in. Support is largely offered through external partners, consisting of community and voluntary organisations, delivering provision from the Life Rooms sites or within the local community. Alongside a pathways advisor, users of the Life Rooms co-create their own social prescription, which typically includes selecting courses from the Life Rooms learning offer (e.g., arts-based activities) or options provided by partner organisations such as support with housing, debt, employment and/or physical and mental wellbeing.

The learning provision is another key component of the Life Rooms model, and is based on principles of co-production and education. The Life Rooms provides learning opportunities ranging from topics such as understanding and managing mental health difficulties through to social and creative offerings. With regard to the latter, the Life Rooms works in partnership with individuals who have an interest in a particular area or topic and arts and cultural organisations (e.g., Royal Liverpool Philharmonic) to offer an array of learning opportunities.

To summarise, the Life Rooms brings together social prescribing, partnership working and coproduction into one service facilitated by the NHS. Although collaborative approaches exist in different guises, such models often do not exist within an NHS context. This evaluation explores the Life Rooms model of partnership working spread over in excess of one hundred partnerships with voluntary, community, and corporate sector organisations.

## Methods

### Ethical approval

The project was deemed a service evaluation according to the local NHS Trust Research & Development department and the Health Research Authority decision tool. Approval for the project was obtained from the NHS Trust Research & Development department (Ref: SE 2020–09). Ethical approval was obtained from the University’s Health and Life Sciences Research Ethics Committee (7881; 7930). All methods were carried out in accordance with relevant guidelines and regulations.

### Recruitment and participants

Semi-structured interviews were conducted with Life Rooms staff members (*n* = 10), partner organisations (*n* = 16), and users of the Life Rooms (*n* = 7) between June and October 2020. All Life Rooms staff members and partner organisations were invited to take part via email. Service users who had accessed support from partner organisations between September 2019 and February 2020 were invited to take part via email and the Life Rooms social media platforms.

### Topic guide

Semi-structured interviews with Life Rooms staff members and partner organisations captured the ways in which the Life Rooms is working with partner organisations, where things are going well and where changes or improvements could be implemented to enhance the model. Semi-structured interviews with service users explored their experiences accessing support from partner organisations under the Life Rooms model. All interviews were audio recorded and transcribed verbatim.

### Data analysis

Data were analysed using the thematic analysis procedure outlined by Braun and Clarke [20]. Thematic analysis is a qualitative method that aims to identify and report recurrent themes in data. Informed deductively by concepts within the topic guide, there were some pre-determined areas the researcher wanted to explore. An inductive approach was then used to reflect on unexpected concepts within the data. The codes were synthesised into categories, which were subsequently grouped into themes. The first and third author met throughout the coding process to examine emerging impressions of the data. Findings from each stakeholder group were triangulated.

## Results

The period between September 2019 and February 2020 saw 609 unique referrals to the Life Rooms. There were 3894 referrals for onwards support made by pathways advisors (see Table 1).

**Table 1:** Number of referrals for onwards support made by pathways advisors between September 2019 and February 2020

Referral categories	Number of referrals
Life Rooms learning provision	2201 (57.0%)
Social support in relation to debt and benefits	217 (6.0%)
Social isolation	214 (5.0%)
Physical health	174 (4.5%)
Employment	117 (3.0%)
Housing	95 (2.4%)
Volunteering	40 (1.0%)
Family/caring role	29 (0.7%)
Other	845 (22.0%)
Total	3894

Five overarching themes were identified from the interview data: ‘Quality of partnerships’; ‘Benefits of partnership working’; ‘Facilitators’; ‘Challenges within the Life Rooms model; and ‘Making things even better’ (see Table 2).

**Table 2:** Overarching themes and subthemes

Themes	Subthemes
**Quality of partnerships**	Shared values: holistic approach, stigma reduction and co-production
	Mutual trust and reciprocity
	Regular communication and face-to-face contact
**Benefits of partnership working**	A cost-effective and sustainable way of working
	Broadening opportunities
	The promotion of psychological wellbeing
**Facilitators**	‘Under one roof’
	Staff with a shared vision
	Professional input
**Challenges within the Life Rooms model**	Equality in partnerships
	Capacity to communicate effectively
	Inappropriate referrals
	Insufficient resources
	Threat from Mersey Care expansion
**Making things even better**	Mechanisms for regular feedback
	Key contacts
	Variety of courses

### Quality of partnerships

#### Shared values: holistic approach, stigma reduction and co-production

When forming a new partnership, the Life Rooms staff strive to develop working relationships with organisations that share similar values:

*We look at the organisations and their values and whether those fit with our values and what we believe the Life Rooms is doing or the Trust is trying to do* (LRs 4).

By adopting a holistic approach, the Life Rooms model considers every aspect of an individual, striving to both empower them and to provide opportunities that enable them to move forward into more fulfilling lives. Partners share this sense of social responsibility and believe this contributes to the effectiveness of the Life Rooms:

*The environment they’ve created is about this holistic approach, which I think is fantastic… One of the reasons we were interested working with the Life Rooms specifically was because that was an approach that they were looking to take. They wanted to bring in an expert for each particular area and an organisation that could add value to what they were doing* (Partner 5).

*The fact that they are so effective is because they look at the whole person* (Partner 13).

Partnerships therefore enable the Life Rooms to provide a comprehensive offer, and the presence of partners helps to attract more people into the Life Rooms buildings, whilst also reducing stigma around accessing the service:

*It helps us in attracting more people to the building*… *I don’t think we would be anywhere near as successful as we are without our partners. They really are vital to us. I think it helps us to get away from the stigma of that’s a mental health building* (LRs 6).

*I hope the value of our brand also helps Mersey Care to again encourage people to use the Life Rooms because it is a place where they can start to access music activities that are provided by Royal Liverpool Philharmonic* (Partner 7i).

With regard to the learning provision, all courses are designed to be accessible and inclusive as partner organisations co-create their courses alongside Mersey Care staff and service users. Partners perceive there are benefits for service users of being involved in the co-creation of course content, aims, and outcomes:

*We very much asked the makers when they were designing the activities, and they were all bespoke to the Life Rooms participants and working with the staff there to make sure that what we were delivering was pitched right for the people taking part*… *The sessions are written particularly for these groups and I think all of that is absolutely the key* (Partner 12).

*Involving the service users is also about creating ownership and value and it’s like in anything if people feel that this is something, that the course that they are part of and that they have a say in the direction of it and the output from it, hopefully that will increase their motivation for not only turning up week in week out but actually wanting to then create the best possible result not just individually but as part of the team and seeing the whole group as part of a team because hopefully that’s when a lot of the skills and the positive outcomes will flow* (Partner 7i).

Thus, for Life Rooms staff and partners alike, shared values were underscored as key for effective collaborative working.

#### Mutual trust and reciprocity

Effective partnerships are characterised by mutual trust and respect, and many partners highlighted the longevity of the partnership in relation to the development of trust:

*I think it’s that longevity of relationships. Partnerships don’t just happen in five minutes; they come over time of working with each other*... *I think all partnerships have to work both ways. It’s about mutual respect and good communication. It’s about trust* (Partner 8).

*We’ve built a relationship that is based on developing a shared understanding of one another, a real respect for one another and also which, even in the test of any difficult times, we know at some point particularly over twelve years, things won’t always be perfect, things happen but we’ve got a real level of trust and honesty* (Partner 7i).

Effective partnerships are mutually beneficial. Although the Life Rooms and partner organisations do not derive the same benefits, it is important that both partners derive benefits they feel are of comparable value. Indeed, mutuality and reciprocity emphasise that both the Life Rooms and their partner organisations work together in a way that is meaningful and beneficial to them as well as to the larger shared goals:

*We absolutely see ourselves as a partner organisation because it’s a reciprocal relationship. It’s not just us providing a service and having a service level agreement. We are responsive to each other and learn from each other* (Partner 7ii).

Indeed, ensuring that partnerships are mutually beneficial nurtures partnerships that are sustainable and builds the capacity to continue beyond a specific programme or course. For example, Liverpool Philharmonic have supported high profile anti-stigma and advocacy activities including the Zero Suicide Alliance launch, the Big Brew and other Mersey Care mental health awareness campaigns:

*The relationship goes beyond the actual activity we deliver in Mersey Care settings into other activities that Mersey Care have supported us to design at Philharmonic hall and also we’ve become part of supporting other initiatives such as the campaigns Mersey Care have led on Zero Suicide Alliance or the Big Brew and other things where we involve our artists. We are an employment partner as well where we guarantee interviews for Mersey Care service users in job applications and things like that. We’ve developed from a very small ‘we will provide a music service to you’ into a real sense of collaborative relationship over the years* (Partner 7i).

Last, sharing information is a further example of reciprocity and is vital to effective partnership working. Some partners provide regular feedback to the Life Rooms about service users’ progress, and this is seen to be an important aspect of partnership working from the perspectives of the Life Rooms staff and partners:

*We agreed that on a monthly basis, we would let them know where we were up to with the customers, we would let them know any progress that was made, we would let them know about the engagement levels... It’s been that sharing of information, which I think has gone really well* (Partner 5).

*I would say that the strong working relationship with them has been due to the fact that we know them really well and they know us really well but also there’s that reciprocation of us sharing information with them and them sharing information with us* (LRs 8).

Taken together, Life Rooms staff and partners cited the importance of fostering trust and mutual understanding of each other for effective collaborative working to take place.

#### Regular communication and face-to-face contact

Effective partnerships seem typically to be characterised by clear, efficient and regular communication:

*Regular communication is absolutely critical and we are very open and transparent with Mersey Care because it’s been that long-term partnership so we can be really open and reciprocal and honest with each other* (Partner 7ii)

*They’ve been very clear in explaining how we communicate with them. That’s been a very important aspect of the relationship and a pretty successful one* (Partner 5)

Communication between partner organisations and the Life Rooms is often enhanced when partners utilise the Life Rooms sites to deliver their support:

*I think having them in the building makes a massive difference. I think them seeing us every week, getting to know our names and faces, I think that in itself really helps* (LRs 8).

Thus, having a physical presence within the Life Rooms environment promotes positive relationships with Life Rooms staff. As face-to-face contact enables efficient and personal communication, key personnel from each organisation have got to know each other on a personal, as well as professional, level:

*I think the key workers who operate in there have got very good relationships with the staff on a personal level, they’ve got a very good professional relationship, and they are able to refer across to each other a lot easier*. *When I go to visit staff in there,* v*ery often the pathways advisors at the Life Rooms will be coming over and talking to me about ‘I’ve got a potential referral here for you, what do you think about them’? Really trying to gain an understanding of our programme* (Partner 5).

In summary, the development of relationships, understanding each other’s offer, and getting to know each other on a personal, as well as professional, level were underscored as key in delivering shared outcomes. Thus, successful cross-sectoral partnership working relies on communication and relationships.

### Benefits of partnership working

#### A cost-effective and sustainable way of working

Partnership working reduces duplication of effort, which is beneficial for both the Life Rooms and their partners:

*Keeping the cost down for both sides. Say we are referring someone to get support with a benefit application, well that individual is already in existence so we don’t have to train an individual within our own service. Likewise for them, if they’re working with someone who is struggling with their mental wellbeing, then they know they can refer straight into the Life Rooms, they don’t have to do anything or train anyone else or waste their resources when those assets are already in the community so it’s time saving, it’s cost efficient, it’s beneficial* (LRs 3).

As the Life Rooms are providing a model to enable smaller organisations to continue operating, this approach adds social value at different levels by contributing to the wellbeing of individuals, communities, cultural activity and society. Thus, there are clear benefits of working in this way for partner organisations:

*Third sector organisations might not have the money for meeting space so we can work together and maybe give them a space for free in one of our Life Rooms to help their organisation keep running* (LRs 3).

There are also benefits of partnership working for the NHS as it may reduce pressure on clinical services:

*If the social issues were addressed, it would take a lot of strain off the mental health sector/the clinical sector so hospitals and clinical services* (LRs 7).

*The benefit for Mersey Care is if you can intervene earlier and prevent or provide effective early support for people, hopefully it will either get rid of the need for them to go into some of the more clinical NHS settings or it will keep people who have recovered and come out of inpatient settings, actually will keep them supported living in the community and independently as well* (Partner 7i).

In light of this, some partners suggested that the Life Rooms model should be implemented on a national scale, as other NHS trusts across the country would benefit from the introduction of this approach in the longer term:

*If every NHS trust could have something like the Life Rooms to try and alleviate problems before they happened, I think it would be a real positive… If that could be the strategy countrywide, I feel like there would be an investment there in the short-term but I feel that in the long-term, there would be money saved there because you would have alleviated a lot of these issues* (Partner 9).

*We work with other NHS organisations in different parts of Liverpool City Region… Wow how life would be so much more straightforward if they had Life Rooms. You can see many of them are talking about similar types of things but I always just say to them ‘just phone [name] and ask him for the blueprint’ because clearly Mersey Care have got a really good model going. Surely it will have some sort of benefit for the NHS* (Partner 7i).

This subtheme illustrated that working with the Life Rooms is a cost-effective and sustainable way of working for partner organisations. As a model for effective cross-sector collaboration, the Life Rooms innovation could provide a blueprint for similar collaborations in other regions of the UK.

#### Broadening opportunities

Crucial to partnership working is developing a shared sense of opening opportunities to make service users’ lives more worthwhile. As many individuals who access the Life Rooms are from disadvantaged communities, the Life Rooms provides access to opportunities that may not otherwise have been accessible to them:

*The creative work with the Playhouse or the music with the Liverpool Philharmonic, it opens up new avenues to people because they are quite rare opportunities for the majority of people, especially the people who use the Life Rooms who might be quite deprived in some ways and might not have those kinds of opportunities* (LRs 9).

Following completion of courses at the Life Rooms, service users are encouraged to access programmes in their local community. Partnership working facilitates this transition as many partner organisations have programmes running in the community that Life Rooms users can attend:

*In terms of activating people moving on from the Life Rooms, that’s where partners come in. You want people to be accessing the community and using what they've learnt on your course [to] then go and live their life* (LRs 9).

*I think the Life Rooms really encourages partnerships because they see their members as being with them for a while but also they want their members to move on and be involved in community activities outside of the Life Rooms* (Partner 8).

*Clients of the Life Rooms then get referred to other courses that we run at Philharmonic hall, specifically for people experiencing mental illness* (Partner 7i).

Community provision provides a bridge to independence, which is important for prevention and long-term recovery. Overall, as the Life Rooms model encourages people to move on following completion of courses in the supportive Life Rooms environment, partners praise the Life Rooms for adopting this approach to developing autonomy and providing pathways for people to engage in their community:

*I think their attitude to their participants is brilliant… What they see is organisations doing other things enhances people’s experiences and actually it’s brilliant if someone moves on to do something else because it means that they have moved on and progressed and they are on a really good path* (Partner 8).

Pulling this together, many partner organisations shared their experiences of the Life Rooms model of partnership working as one that broadens opportunities for communities. Partnership working enables people with mental health difficulties to optimise their health and enrich their life experience in the broadest sense possible.

#### Promotion of psychological wellbeing

Creative courses facilitated feelings of accomplishment and achievement because service users developed their artistic abilities, began to believe in their skills and gained a real sense of satisfaction:

*Who would have thought I can sing? We are sounding harmonious. It’s incredible. Again it’s that ability of someone to bring out the best in you, to awaken something in yourself that either you never thought you had or you were convinced you didn’t have or you compared badly to someone else* (SU4).

Courses facilitated by some of Mersey Care’s cultural and creative partners such as Liverpool Philharmonic and Liverpool Everyman and Playhouse culminate with a live performance. As these venues are places that may have previously felt inaccessible, service users appreciate the opportunity to perform in such prestigious venues. Such opportunities may enhance feelings of belonging, sense of mastery and personal growth:

*To go to the Playhouse and to be in that that atmosphere doing a performance instead of in the Life Rooms gives it more atmosphere* (SU7).

*Every one of us felt that being part of a bigger organisation that was dedicated to this activity at the Philharmonic and there is a little bit of prestige about it as well to say I stood on the stage at the Philharmonic* (SU4).

Furthermore, attending courses at the Life Rooms removes barriers to social inclusion, and attendance becomes part of people’s on-going social life. Creative pursuits improve inclusion as such activities enable individuals to share an experience together, which benefits wellbeing by enhancing togetherness. Courses create a shared interest, which facilitates connections and development of friendships:

*It’s not always going to be the actual music itself that produces the outcomes, it might be just that shared experience of working as part of a team* (Partner 7i).

*I think for the participants, the service users, the benefits would be that a lot of them have made friends with each other now and they go out shooting photography together* (Partner 9).

When reflecting on their Life Rooms experience, some service users found engaging in creative activities to be ‘life changing’ and referred to such activities as ‘a therapy outside the hospital’:

*Life Rooms has been life changing but the thing that changed our lives was having people [referring to partners] not focus on our depression but focus on our new interests and focus on them with people [partners] who really have a passion for those interests* (SU4).

*In the past, my experience with Mersey Care was being a patient in hospitals and occupational therapy and this [referring to a poetry course] is like a therapy outside the hospital* (SU5).

To summarise this section, the meaningful activity and authentic engagement offered through such courses are vital in promoting wellbeing and encouraging a sense of hope. Creative and cultural courses act as a catalyst for promoting change.

### Facilitators

#### ‘Under one roof’

The Life Rooms is an all-encompassing hub, which facilitates easy access to a range of different courses and resources provided by partner organisations:

*Partners provide us with services as well so they come into our buildings and facilitate sessions or provide services from our buildings. It's part of linking the community together because it puts everybody in one place. It’s easily accessible for everyone* (LRs 9).

*Ordinarily somebody might come to you for an appointment if they’ve got problems with their benefits, if they’ve got problems with their housing or they might want to get into employment, and traditionally you would be passing them from pillar to post… We’re saying to them if you come into our service, as best as we possibly can, we will meet your needs in this space if you can get to us our partners are there* (LRs 3).

Providing an array of services ‘under one roof’ is beneficial as it is common for people experiencing mental health difficulties to find attending services or courses in unfamiliar locations challenging:

*[The Life Rooms are] able to organise those kinds of meetings for you to have on the premises with somebody who comes in from outside, and that was also very useful and supportive at a time when… my mental health at that stage was not managing to cope with much in the way of decision making or trying to actually navigate my way around*… *I couldn't have envisaged going to a class where I went to find it myself and I went along. That would have been far too daunting* (SU1).

Thus, partnership working is beneficial as Life Rooms service users are offered an appointment with an external service in a familiar setting that they feel comfortable in. According to partners, feeling comfortable in this space also enables service users to engage with their creative courses:

*I think it’s because Mersey Care have done an amazing job in creating a place that’s a safe space that doesn’t feel like a hospital setting but equally it’s got that wraparound care and support of the NHS and I think it’s remarkable what they’ve done and how they’ve set up the spaces is fantastic. It makes it therefore an environment that’s conducive to people not being on edge and to feeling comfortable, to feeling ok to have fun and to make a bit of noise and to sort of feel safe but equally to just relax and throw themselves into an activity as well* (Partner 7i).

Data falling under this subtheme illustrated that the Life Rooms is an all-encompassing hub, which facilitates easy access to many resources and support opportunities. For service users, having the resources in one place where they can access a number of different support opportunities is beneficial.

#### Staff with a shared vision

Delivering sessions within the supportive Life Rooms environment is beneficial for partners as it means that there are experienced staff on hand should they encounter any issues during their sessions:

*It’s also a safety net for some people should something come up in my sessions, and sometimes it does, it triggers something for somebody or someone discloses something, there is help at hand… I think it’s really important that working within that space, that’s all on hand* (Partner 8).

This is also beneficial for the Life Rooms as it provides a space for staff members to acquire new skills with the aim of delivering similar sessions in the future:

*There’s always a member of Life Rooms supporting and sometimes it’s their mentoring staff so people who have previously been service users and have now started to volunteer and help support some of the activities that the Life Rooms delivers. We feel like we pass skills on to them* (Partner 12).

Many partners highlighted the importance of having a key point of contact in Mersey Care who is familiar with their organisation, in charge of the day-to-day communication, and the resolution of any queries or issues:

*Having that member of the team in [name] who’s in Mersey Care, who understands how it all works, knows all the people, is incredibly well respected and is such a lovely person to work with, actually that just saves so much time and energy and effort… It means we’ve got access to that expertise that we can just run things by, talk through things, test ideas out, get an opinion on, which is really effective* (Partner 7i).

*It just makes the world of difference when you’ve got somebody who you know you can just reach out to and get advice from or ask for some information with regard to Life Rooms. It definitely helps to have a key contact* (Partner 14).

Overall, partners highlighted the importance of supportive and passionate staff. Staff buy-in is essential if the relationship is to be successful and sustainable in the long-term:

*There’s a real, it just jumps out at us about the value the Life Rooms teams place on the work that we do as well as the service users. That’s so important to us because we like to be in long-term partnerships that can develop together where the other partner actually wants us to be there*… *I think the enthusiastic and the commitment of the Mersey Care staff that work with our musicians is fantastic and a real willingness to get stuck in and to get involved and to understand it and to work with the musicians. That real sense of professional respect and collaboration between musicians and Mersey Care staff is absolutely at the heart of it* (Partner 7i).

Taken together, it is important for Life Rooms staff and partners to share the same vision and sense of purpose. Feeling respected and respecting others were also underscored as key in delivering shared outcomes.

#### Professional input

As pathways advisors do not have knowledge and expertise in all areas, joined-up working alongside a vast array of external partners is beneficial:

*I just think our job is so much more enriched for being able to offer so many different organisations and the scope of help is just massive and the fact that each partner will be a specialist in their own field so they know exactly what they’re talking about* (LRs 5).

With regard to the learning provision, courses are delivered by highly skilled professionals such as musicians from Liverpool Philharmonic hall or professional craft makers from Bluecoat. As delivery from a highly experienced team results in high quality delivery, the added value of professionals delivering courses within the Life Rooms community hubs was highlighted:

*I think the key to our whole outreach programme is that we use professional working makers... It’s a high quality delivery. I absolutely believe that is the key to success. It’s something that’s very carefully considered and the sessions are written particularly for these groups* (Partner 12).

*I think the thing that was most prominent for me was the quality of the external involvement. It far surpassed anything I was expecting… There’s a difference between going with somebody who is just interested in it and having somebody whose life interest is to work in that area and to share their passion and share their interest. It’s wholly different. There’s quite a difference in what you can get out of it* (SU4).

Given their wealth of experience, partners are able to tailor their bespoke courses. For example, Liverpool Everyman and Playhouse staff tailored their storytelling course, whilst musicians from Liverpool Philharmonic often respond to the mood of the group using music:

*The musicians then respond quickly to the mood and the discussion that’s happening as well. They might completely change the piece of music they were about to play in 30 seconds based on a conversation that’s happening between the service users in the session* (Partner 7i).

*She seemed to think that myself and another participant were a little bit beyond the maybe beginner level of what the course was aimed at, and so because there was really only myself and this other guy attending, she really tailored it to us. It was just wonderful. It was a real gift on a plate for me given what I was going through at the time emotionally* (SU3).

Thus, partners can be flexible and responsive to the needs and interests of those who participate while retaining the structure and objectives of the sessions they deliver:

*They're probably quite traditional writing workshops but the actual design of them could change according to what people would like to do so I’m flexible in that sense. I don't stick to a rigid way of doing this. Say for example, someone brought a photograph in or they brought something in of interest that could change the nature of the workshop… And I might then design the next one to be slightly different. It's really about keeping people engaged, interested and very involved really as it’s for them* (Partner 15).

*I think our musicians certainly respond to service users, they involve them in the development of the courses as they are progressing and we get their feedback to make sure that it’s feeding into what we do* (Partner 7i).

In sum, service users acknowledged the added benefits associated with attending courses delivered by highly skilled professionals. Some service users valued the quality of external involvement, particularly acknowledging the importance of quality and how this makes a difference to their experience.

### Challenges within the life rooms model

#### Equality in partnerships

When developing a new partnership, it is important to ensure that the needs of both organisations are considered. However, some partners perceive a lack of equality as Mersey Care is seen to be the dominant partner:

*There is not that kind of equality really between the partners and the organisation. To develop a culture of that would be great* (Partner 15).

One partner found the partnership agreement form to be one-sided in favour of Mersey Care, thus highlighting the issue of inequality from the outset:

*One of my staff was approached by a pathways advisor to sign a partnership agreement which I had a look over and it was very one-sided weighted in the favour of Mersey Care*… *It was very much a large service provider’s partnership agreement. It didn’t really think through from a charity’s perspective or a partner’s perspective. It’s all very from a Mersey Care angle* (Partner 6).

In order to achieve best practice collaboration, the Life Rooms need to address such perceptions, as partnerships perceived as unequal may not sustain. One partner suggested that such issues ought to be addressed in face-to-face meetings:

*Rather than a disembodied referral, that we actually have a physical relationship where we meet each other as equals and we meet around the table and we get a common understanding of what we are doing and why we are doing it* (Partner 1).

In summary, this subtheme illustrated that equalising perceived imbalances between sectors is important in order to sustain partnerships. Learning is required in order to ensure the equity of partnerships, especially as the Life Rooms is part of a large NHS organisation.

#### Capacity to communicate effectively

Due to the array of partners, Life Rooms staff can struggle to keep communication flowing smoothly. As a result of communication issues, some partners were not aware of the full range of services on offer at the Life Rooms:

*When you’ve got a lot of partnerships that you’re working with, mental wellbeing, physical wellbeing, housing, employment but within each of those categories there are numerous partner organisations and signpost organisations that we use and actually trying to keep in touch with all of those organisations is extremely difficult* (LRs 8).

*I think the only barriers were just knowledge or information. I suppose like not knowing the full range of the services offered. I think that’s been our only barrier so we wouldn’t have referred in for vocational training or employment support necessarily because we didn’t know that they offered that* (Partner 2).

Other examples of communication issues include lack of both formal and informal catch-ups:

*According to the SLA [service level agreement], we were meant to have regular catch-ups, quarterly meetings, they tended not to happen. There were communication issues* (Partner 6).

*No one comes in and says ‘how’s it going?’ It’s all very busy… There is no debrief or I get no feedback from the Life Rooms* (Partner 10).

Communication issues have filtered into service users’ experiences, with one service user suggesting that communication between partner organisations and the Life Rooms could be improved:

*I think for communication just to be a bit quicker and a bit slicker between the two would really enhance things... I suppose it's communication at the administrative level that perhaps could help, could be improved and that would help to really kind of streamline experience for everybody* (SU3).

Taken together, ongoing formal and informal communication and networking opportunities would be beneficial moving forward.

#### Inappropriate referrals

The bidirectional referral of individuals between the Life Rooms and partners can feel inappropriate at times:

*A lot of partners can send inappropriate referrals through. One of the main difficulties is when people are quite unwell, quite poorly, who actually need more secondary care intervention and they get sent through to us before they have been sent through to secondary mental health care. With that, it gives us quite a lot of work to do* (LRs 3).

*We do sometimes get what we might term as inappropriate referrals so maybe people with multiple barriers that need sorting before they are work ready* (Partner 16).

As different organisations have different capacity levels, pathways advisors need to ensure that they do not inundate smaller voluntary organisations with a high volume of referrals. Similarly, pathways advisors also need to consider whether specific individuals are eligible for support from each partner organisation that they refer people to:

*A small third sector organisation that we might think is amazing and offers great support for our clients, they might only have three staff. If we are shooting referrals at them, it might take them a while to pick that person up and that’s not any fault of their own. It’s just because they haven’t got the capacity so we have to be really careful not to inundate the third sector organisations as well and make sure we aren’t being detrimental to their service by the types of people we’re sending through, the issues that people might have* (LRs 3).

*There’s always challenges around because of areas so depending on what postcode they’re in, that can provide some challenges in that they may not be able to access certain support because they are not in the catchment area. There may be challenges around whether they are on benefits or not. Some of the offers that we have are open to people who are on benefits* (LRs 8).

One service user was referred to a partner organisation that was not in a position to support her due to previous service usage:

*They put me in contact with them but Sefton at Work obviously can't help me because I was with them three years ago and they only have an amount of funding for each individual* (SU2).

In sum, there were incidences of partner organisations referring into the Life Rooms inappropriately as well as incidences of partner organisations receiving inappropriate referrals which often causes challenges. This highlights a clear communication issue around identity of the different services. Partner organisations would benefit from targeted information on the Life Rooms model to enable them to feel more confident making referrals.

#### Insufficient resources

Some partners have encountered challenges when delivering their provision within the Life Rooms. For example, some partners were allocated rooms that were unsuitable to effectively deliver their sessions:

*When I first went there, they put me in a classroom upstairs. I had three people who couldn’t be evacuated plus two carers as well. I said that room is not suitable. It’s upstairs and there’s not enough room for everybody to sit round. One lady had a panic attack. There was nowhere to teach. There was no board in the room* (Partner 10).

When working with vulnerable people, it is common for issues to arise whilst delivering support or courses at the Life Rooms:

*Sometimes people talk about wanting to hurt themselves or want to talk about things that have happened in their lives and some people might come in and be very vulnerable and out of that vulnerability comes issues that I think hang on a second, I think that needs to just be flagged up with somebody here* (Partner 8).

In light of this, partners delivering courses value the presence of Life Rooms staff members supporting each session in order to provide support if an individual becomes distressed. However, issues with staffing capacity when supporting sessions were highlighted:

*There have been some issues that Life Rooms are aware of with some of our sessions running on Life Rooms sites in terms of appropriate staffing. That’s something we are working with Mersey Care on so making sure that there is the appropriate staffing to support any sessions, particularly if there is somebody there who begins to feel distressed or upset, being able to have someone there who can support them appropriately* (Partner 7ii).

For some partners, teaching in this environment was unfamiliar, and as there was no formal induction process, they encountered difficulties during the first few weeks:

*There’s not a clear induction process if somebody just stepped in to do something. In my experience, that hasn’t happened* (Partner 15).

Thus, partners have identified a prior training need to ensure that people know what to expect when they start working in partnership with the Life Rooms. Each partner should be given a clear induction, and a starter pack would be beneficial for those delivering courses:

*I would have really benefited from a real induction. I was sort of parachuted into something that I wasn’t expecting. I think if they are looking to other people providing the service, most definitely I think that’s really a responsibility of the Life Rooms to say these are our clients*... *You need to do a proper induction… Maybe a little pack or something: ‘If this happens, this is what you need to do and these are the people you need to speak to if you’ve got any concerns’* (Partner 10).

Last, a few participants highlighted funding as an issue. Although there are benefits of having partners physically present in the Life Rooms, some require funding to deliver their services from within the community hubs:

*There are no guarantees that [name] and I will continue to work with the Trust. With NHS funding and the way things are, it’s reviewed every year* (Partner 15).

Funding to enable some partners to continue delivering their provision from the Life Rooms has been lost, and resource implications have filtered into service users’ experiences:

*It [referring to yoga] really made a big difference to me, and very sadly, the Life Rooms had to make the decision, a funding decision, and she [yoga teacher] couldn't continue. Which was a great shame… I know for everybody else that did those classes, how desperately we missed that and how upset we were when they had to end and that was a funding issue* (SU1).

*One of the difficulties when we talked to the staff at Life Rooms about why they haven’t brought the fabulous florist back and they said it’s all down to finances. If their finances are such that another £1 an hour makes such a material difference, then they are spending their pounds quite wrongly. You have to address what’s good. Instead of having them for six weeks, you have them for five consecutive weeks. That’s far better than having somebody do six weeks and the quality of what you walk away with is much poorer* (SU4).

Moving forward, the Life Rooms should provide tailored introduction material for partners so that new partners have a clear idea of the aims of the service and what they can expect from the Life Rooms.

#### Threat from Mersey care expansion

Although the Life Rooms utilise a community-asset based model, some partner organisations regarded Mersey Care’s expansion as a threat. More specifically, partners expressed concerns for small voluntary organisations that may not be able to continue their provision if the Life Rooms provide it as part of their core service offer:

*When they cross over into the community and voluntary sector, into the third sector, I have an issue because that’s the world that I listen to and that’s what I’m hearing back at me, genuine fears of people who have run small little organisations for years saying they are going to do it for us now* (Partner 1).

*There are lots of small organisations and community groups who are doing really good work that are struggling for funding and I think that they are a little bit fearful that Mersey Care are growing up that community offer very very wide and that could be detrimental to a lot of community groups that maybe they could be helping to fund, to support work that they do* (Partner 4).

One partner believes that the Life Rooms competes with the voluntary and community sector:

*There is a danger that if we focus too much on these monolithic providers, we will lose this rich tapestry that Liverpool has. That would be one of my fears anyway. It’s about acknowledging and respecting small voluntary organisations. There are things that are being done in our community that are best done in our community*… *The Life Rooms in my judgement is the part of Mersey Care that is setting itself up as in competition to the voluntary and community sector* (Partner 1).

In particular, partners highlighted the similarities between the Life Rooms model and Person Shaped Support (PSS) wellbeing centres:

*It’s very similar to PSS. PSS have their wellbeing centres and how does the model of the Life Rooms compare to the PSS wellbeing centres? I’d be fascinated to see how that worked* (Partner 15).

*PSS has done fabulous work around art therapies and gardening and different things and the Life Rooms does them now but they do them in a beautiful building, better resources, better this, better everything but they haven’t got the years of experience working in the community that PSS has got* (Partner 1).

Thus, the Life Rooms might consider similar models, such as PSS, as it would be beneficial to look at synergies and working together more closely:

*I’ve been in groups that have been disparaging about Mersey Care in general. I thought to myself it’s the biggest mental health organisation on Merseyside so you’ve got to embrace what’s there as well as maybe campaign to get people to work better without someone taking away their service* (SU5).

To summarise, Mersey Care NHS Foundation Trust is constantly expanding its reach. As the Life Rooms have developed and expanded as well, some partners perceived this as a threat, especially as the Life Rooms learning offer overlaps with provision provided by small voluntary organisations who are struggling to source funding to continue their provision.

### Making things even better

#### Mechanisms for regular feedback

In order to understand the sustainable impact of the model, it would be beneficial for the Life Rooms to routinely collect data from service users to better understand how they have been supported by partner organisations and the effectiveness of the support:

*Sometimes we don’t get information from the clients about a partner organisation… They could say yes that organisation really helped me or no I didn’t feel like I got the help that I needed from that organisation and therefore I need to go back or I need another organisation to help support me* (LRs 8).

Similarly, regular feedback from partner organisations would be useful in terms of developing and improving the service:

*I think if we get more feedback from them [partners], then we can obviously continue to develop as a service to help support them as an organisation. I think that’s key* (LRs 8).

*What I think would be great for our service is for the partners to have more of a say in how we are working in the Life Rooms* (LRs 3).

Thus, it may be beneficial for the Life Rooms to hold more partner events in order to gather feedback from partners. This would enable partner organisations to network with one another:

*What we have lacked so far due to capacity and time has been getting all the partners in the room and having a bit of a reflection. Let’s have some ideas. Let’s see how we can develop the service* (LRs 3).

*The problem is, creative partners don’t get many, well there aren’t any forums to talk to each other*… *It could be done in lots of different ways but I think twice a year would be good. Not necessarily pressure for everybody to get up and do a big presentation but just to share progress and some discussion of what’s worked really well because unless I look it up or find out about it, I’m not going to know what other partners are doing* (Partner 15).

Moving forward, partners would value ongoing networking opportunities to provide feedback on the Life Rooms model and facilitate connections with other partner organisations. Partners would also welcome transparency around each other’s offer.

#### Key contacts

Not all partner organisations are assigned a key contact within Mersey Care. Partners who do not have a key contact would find this beneficial in order to prevent re-explaining their service offer to different staff members:

*The only challenge I would say was that I’ve had three different people to deal with… That’s the only challenge I suppose having to explain myself every time. I suppose make sure we have a dedicated person. So you have the same person each time* (Partner 3).

Similarly, keeping key members of staff from partner organisations consistent also has real added benefits:

*[name] who works at Walton with us, she’s basically been the point of contact for the best part of four years now so we’ve managed to keep that relationship consistent so that everyone knows who they are speaking to so I think that that has been a massive help as well* (Partner 5).

*It’s not always easy to communicate clearly between different organisations because you always start off with two or three people from each side involved but then it might escalate to we need to talk to finance or we need to talk to this person or that person so then before you know it there are quite a few people involved and you lose track of who is doing what… I think it is just about having some really clear communication with key people* (LRs 10).

However, often pathways advisors do not know individuals from the partner organisations who they are referring service users to for support:

*That in itself can be one of the challenges because for a lot of organisations, you don’t actually know who you are referring the person to and they don’t know who you are* (LRs 8).

*We don’t know who people are, they are less involved, they are less present. We receive referrals but there is no development of relationship* (Partner 1).

To summarise, the development of relationships and having a key contact would enable the process of collaborative working to operate smoothly.

#### Variety of courses

Partners and service users highlighted that although the Life Rooms place a large focus on their practical workshops, it may be beneficial to provide a more balanced offer:

*I’d just like to see a lot more of it really and more focus being given to maybe the creative side than the medical self management side. I’m not saying that doesn’t matter. It does. But I’d like to see an equality of approach in terms of creativity* (Partner 15).

*I felt very strongly was that there was a slight imbalance between the mind body connection and that there was a lot of more practical aspects about coping and management. So managing anxiety, managing your finances, these kinds of things… I did feel strongly, particularly after the funding that was lost for the person for yoga, that there wasn't many things like meditation, relaxation, yoga, all of the kinds of things that you do, which are mindful to improve your physical connection with mental state was not as well served* (SU1).

Partners would also welcome the opportunity to deliver courses in collaboration to further support the idea of a network supporting people holistically in non-medical ways:

*I think that would be great especially if it linked up some people together. I did one workshop, only once, it was in Broadoak and [name] was there and it was just serendipitous. We actually worked together and we hadn’t planned anything but we did one workshop and I was doing some poetry work and [name] was there and she played in response to stuff and that was just I mean it was a one off but it would be fabulous* (Partner 15).

*I’ve spoke to [name] who runs a monthly writing class there and we had a small discussion about potentially rolling the writing class into the photography class one month because I think there is a bit of a cross-over between writing and photography* (Partner 9).

Moving forward, partners could be given the opportunity to offer creative courses in collaboration with each other to further extend the Life Rooms model as a network supporting people holistically.

## Discussion

The Liverpool City Region has a richness of voluntary organisations and community based assets that the Life Rooms innovation aims to draw upon to widen the base of health and wellbeing support and to include the full participation of arts, voluntary and community organisations in the local health economy. In line with UK policy developments [6–8], this joined-up approach between health, community, voluntary and arts sectors considers every aspect of an individual, empowering them and providing opportunities that enable them to move forward with more fulfilling and meaningful lives. Consistent with previous research [11], working in partnership delivers high quality care by supporting people holistically, and by collaborating with voluntary and community organisations to do so, the Life Rooms model is essential in shifting the traditional care model. As a network supporting people holistically in non-medical ways, successful partnership working under the Life Rooms model appears inclusive and accessible, responsive to service users’ interests and needs, and involves collaborative working with co-designed content, aims and outcomes.

The Life Rooms have developed over 100 partnerships with statutory, private and voluntary sector organisations to enable people with mental health difficulties to optimise their health and enrich their life experience in the broadest possible sense. The Life Rooms offers a service model that promotes the role of the wider system, and therefore allows people to access support that addresses their needs. As this non-clinical approach provides integration of public, private, voluntary and community sector services, the end user does not have to navigate the complexities of a heterogeneous system and instead experiences a seamless pathway of advice, support and care [12]. Although community collaboration exists in different guises, such models often do not exist within an NHS context. The Life Rooms is therefore an exemplar model for the ways in which community organisations can work with an NHS organisation to support local communities.

Collaboration between multiple sectors and the vast array of external partners enables the Life Rooms to offer health-focused, practical/social and arts-based opportunities all within one service. Many partner organisations deliver services from the Life Rooms community hubs to provide support ‘under one roof’, which facilitates easy access to a range of different courses and resources. The bringing together of such a wide range of opportunities for advice, support, learning and self-development within a non-clinical environment is an innovative approach [13]. Enabling partners to offer their services or facilitate courses from the Life Rooms community hubs was found to be effective. The presence of partners helps to attract more people into the Life Rooms buildings, whilst also reducing stigma around accessing the service. In addition, travelling from one place to another for support can be difficult when experiencing acute distress. Consistent with previous research (e.g., [21]), accessing a number of different resources and support opportunities in one location is beneficial for service users.

The integration of private, public and voluntary sectors has the potential to improve outcomes for individuals [13]. The present findings demonstrate that users of the Life Rooms experienced growths in confidence and self-worth alongside fostering relationships with similar others. Crucial to partnership working is developing a shared sense of opening opportunities to make service users’ lives more worthwhile. As many individuals who access the Life Rooms are from disadvantaged communities, the Life Rooms provides access to opportunities that may not otherwise have been accessible to them. Our findings demonstrate that service users’ lives are improved through becoming involved in communities of geography, practice or interest. Working with partners also provides pathways to independence, removes barriers to social inclusion, and facilitates better health and wellbeing. Partnership working provides a bridge to independent activities as many partners offer community provision, which Life Rooms members can access. For many service users, partner organisations provide a sense of belonging as attending courses enables them to share experiences with similar others whilst feeling part of an arts organisation such as the Royal Liverpool Philharmonic. Thus, partnership working is one of the greatest vehicles to enable a fuller mission around psychological wellbeing.

Consistent with previous research [9, 10], the key principles of partnership working are mutual trust and respect, shared values and goals, and regular communication and contact between partners. Our findings, in concert with existing studies (e.g., [12]), demonstrate the importance of fostering trust and mutual understanding for effective collaborative and partnership working. The development of relationships, collegiate values and understanding each of the sectors were underscored as key in delivering shared outcomes. The themes of mutuality and reciprocity highlight that the Life Rooms and partner organisations work together in a way that is meaningful and beneficial to themselves as organisations as well as to larger shared goals. Ensuring that partnerships are mutually beneficial nurtures partnerships that are sustainable and builds the capacity to continue beyond a specific programme or course. Mersey Care NHS Foundation Trust has nurtured a number of creative and cultural partnerships in such a way that these partnerships have now become integral elements of the Life Rooms offer. Creative and cultural partners have a single point of contact within Mersey Care NHS Foundation Trust who is familiar with their organisation, in charge of the day-to-day communication, and the resolution of any queries or issues. Creative and cultural partners highlighted this as a key factor in enabling the process of collaborative working to operate smoothly. Indeed, having a single point of contact eliminates miscommunication, and prevents the repetition of information.

Despite the perceived benefits, there are a number of ways in which the Life Rooms model of partnership working could operate more effectively, and more like an equitable network. In order to understand the sustainable impact of the model, it would be useful to seek feedback from service users who have been supported by partner organisations in relation to their practical or physical needs. Feedback should be routinely collected in order to gather information around the effectiveness of support provided by each partner organisation. Similarly, it would be beneficial to hold regular events for all partners in order to provide opportunities for partners to provide feedback, discover each other’s offer, and create cross-sector pathways of support. Partners could also be given the opportunity to deliver courses in collaboration to further extend the network supporting people holistically in non-medical ways. Last, as many small voluntary organisations are struggling to source funding to continue their usual provision, some partners expressed concerns around the replication of services provided by small voluntary organisations. As the learning offer at the Life Rooms overlaps with some of the activities that small voluntary organisations offer, partners suggested that, rather than replicating services that already exist in the community, the Life Rooms should consider similar models, such as PSS, as it would be beneficial to look at synergies and working together more closely.

This research has limitations. First, although the Life Rooms has over one hundred partnerships with voluntary, community, and corporate sector organisations, only sixteen partner organisations participated in the present evaluation. Thus, the findings are limited to those partners and may not be representative of the views held by all partners. Similarly, as only seven users of the Life Rooms participated in the evaluation, the views from these individuals may not be representative of the experiences of all service users who have been supported by partner organisations under the Life Rooms model. Although the perceived cost benefits of working in this collaborative way have been demonstrated, more robust research exploring the cost effectiveness of this approach is warranted. Indeed, further research into the wider benefits of partnership working on health services would be beneficial. This would support the implementation of this model on a larger, national scale.

Overall, the unique Life Rooms model has many positives. Although some partners expressed concerns around replication of the voluntary and community sector, the Life Rooms provides a framework that is streamlined, enabling people to move smoothly between health and community support and it has successfully shifted the traditional view of mental health support. It delivers bespoke joined-up working between the health sector, social sector, the arts, voluntary and community sectors. The success of this approach illustrates how multiple bodies play a role in supporting health, bringing a blend of diverse skills, expertise and programmes together to support individual mental health and wellbeing. To conclude, as forming partnerships with external organisations engenders more effective service provision, the Life Rooms innovation could provide a blueprint for similar collaborations in other regions of the UK.

## Data Availability

Qualitative data extracts are presented in the article to support the findings. The data generated and analysed during the current study are not publicly available as the data collected is sensitive and could compromise the confidentiality and anonymity of the participants but are available (limited) from the corresponding author on reasonable request.
